# Utilisation of Quartz Crystal Microbalance Sensors with Dissipation (QCM-D) for a Clauss Fibrinogen Assay in Comparison with Common Coagulation Reference Methods

**DOI:** 10.3390/s16030282

**Published:** 2016-02-24

**Authors:** Stephanie Oberfrank, Hartmut Drechsel, Stefan Sinn, Hinnak Northoff, Frank K. Gehring

**Affiliations:** Biosensorik-Gruppe, Institut für Klinische und Experimentelle Transfusionsmedizin (IKET), Universitätsklinikum Tübingen, Otfried-Müller-Str. 27, 72076 Tübingen, Germany; stephanie.oberfrank@freenet.de (S.O.); hartmut.drechsel@web.de (H.D.); s.sinn@gmx.net (S.S.); hinnak.northoff@med.uni-tuebingen.de (H.N.)

**Keywords:** Quartz Crystal Microbalance with dissipation (QCM-D), Clauss Fibrinogen Assay, Blood Coagulation, Blood Viscoelasticity, Fibrinogen

## Abstract

The determination of fibrinogen levels is one of the most important coagulation measurements in medicine. It plays a crucial part in diagnostic and therapeutic decisions, often associated with time-critical conditions. The commonly used measurement is the Clauss fibrinogen assay (CFA) where plasma is activated by thrombin reagent and which is conducted by mechanical/turbidimetric devices. As quartz crystal microbalance sensors with dissipation (QCM-D) based devices have a small footprint, can be operated easily and allow measurements independently from sample transportation time, laboratory location, availability and opening hours, they offer a great opportunity to complement laboratory CFA measurements. Therefore, the objective of the work was to (1) transfer the CFA to the QCM-D method; (2) develop an easy, time- and cost-effective procedure and (3) compare the results with references. Different sensor coatings (donor’s own plasma; gold surface) and different QCM-D parameters (frequency signal shift; its calculated turning point; dissipation signal shift) were sampled. The results demonstrate the suitability for a QCM-D-based CFA in physiological fibrinogen ranges. Results were obtained in less than 1 min and in very good agreement with a standardized reference (Merlin coagulometer). The results provide a good basis for further investigation and pave the way to a possible application of QCM-D in clinical and non-clinical routine in the medical field.

## 1. Introduction

Human haemostasis is a very complex and meticulously balanced system that requires sequential enzymatic activation and smooth cooperation of a set of different coagulation parameters. Fibrinogen (Coagulation Factor I) is a major plasma protein and marks the crucial end point of the coagulation cascade. Conversion of fibrinogen to fibrin followed by its polymerization results in formation of a mashed and insoluble clot which stops bleeding. The reference range level of fibrinogen in humans lies generally between 1.5–4.0 g/L [1]. The assessment of fibrinogen levels plays an important part for medical diagnoses, treatment and screening. Besides prothrombin time and partial thromboplastin time, fibrinogen is the third most frequently assessed non-cellular coagulation parameter in medical laboratories and serves routinely as a diagnostic tool for global assessment of coagulation function [1]. For the management of trauma and emergency patients as well as during extensive surgical interventions it can be of utmost importance in clinical settings to be able to assess and monitor the coagulation status promptly and continuously [2,3]. Furthermore it is important to facilitate the screening and supervision of fibrinogen-related diseases and their therapy, especially in non-clinical settings where medical laboratories are not available, as elevated fibrinogen levels are associated with cardiovascular disorders like coronary heart disease and stroke [4,5,6,7] and fibrinogen defects (e.g., hypo- or hyper-fibrinogenemia) can cause severe conditions in patients. Clinical and non-clinical context both strongly point to the benefit of handy-sized quartz crystal microbalance with dissipation (QCM-D) based assessment devices, which can be operated easily, provide results as promptly as possible, conduct measurements independently from sample transportation time, laboratory location, availability and opening-hours and therefore perfectly complement routine diagnostics.

There are a number of different assays for the measurement of fibrinogen levels in human blood plasma, although the most common method in routine use is the functional Clauss fibrinogen assay (CFA) [1]. For this purpose 1:10 diluted plasma is mixed with a high concentration of the enzyme thrombin and the clotting time is measured either mechanically or turbidimetrically. Thrombin cleaves soluble fibrinogen to fibrin which then can polymerize to form the insoluble, gel-like clot. Said in technical terms, it changes the viscous/viscoelastic properties of the mixture. *In vivo* the inclusion of platelets further stabilizes the clot. The clotting time of the CFA is referred to as the time it takes for the coagulation clot to form and to exceed the threshold of a certain optical or mechanical density. Due to the addition of excess amounts of thrombin, the assay is independent of the thrombin concentration, with the clotting time being inversely proportional to the amount of fibrinogen in the sample. For proper evaluation a calibration curve is established by preparing a series of dilutions (1:5–1:40) using reference plasma to give a range of fibrinogen concentrations. The clotting times of these dilutions are measured and the results plotted in a logarithmic graph.

Due to the fact that QCM-D sensors, in addition to changes in surface attached mass, are also sensitive to changes in viscosity and viscoelasticity, the QCM-D method is nicely applicable for blood coagulation measurements like the assessment of fibrinogen levels. QCM sensors are oscillatory transducers. Applying an alternating voltage to electrodes placed on both surfaces of the quartz generates a characteristic standing mechanical shear wave within the quartz sensor. The generated characteristic resonance frequency f_0_ and dissipation signal Γ_0_ are monitored and recorded. Coagulation-induced QCM signal changes can be monitored in real time during clot formation. Subsequently, the evoked frequency and dissipation signal shift can be used for interpretation and evaluation. In accordance with the Sauerbrey equation [8], mass changes of thin rigid film layers on sensor surfaces are directly proportional to the change of their induced resonance frequency (∆f ~ ∆m with ∆Γ = 0 Hz). The signals according to wetting of the surface with a purely viscous liquid (a Newtonian liquid) is described by the Kanazawa equation [9]. For viscoelastic and complex multilayer films, such as e.g. bio-layers and blood coagulation processes, the presented mathematical models for signal description and analysis had to be further adapted [10,11,12,13,14,15]. Therefore, advanced QCM-D sensor setups like the one used in this work are not only suitable to record changes in mass on a nanogram scale but also changes in dissipation and hence to physical properties such as density, thickness and viscosity. Thereby the dimension ∆Г is proportional and concordant to D, the reciprocal of the quality factor Q, as used by [11].

Recognising the potential of QCM sensors for coagulation measurements led to an increased scientific interest in different types of QCM-based blood and coagulation experiments over the last decade. Especially experiments in regard to rheological characterization and modelling of blood and blood coagulation [16,17,18,19], platelet aggregation [20,21,22,23], thromboplastin time [22,24], activated partial thromboplastin time (aPTT) [25], changes in viscoelasticity [18], multiparametric assessment of blood coagulation processes [26,27] as well as surfaces activating and inhibiting coagulation [28,29,30] have been conducted since then. Only recently have the use and suitability of QCM sensors for the qualitative and quantitative measurement of fibrinogen come into focus. In comparison to this work, publications with QCM based focus on fibrinogen mainly follow up with detection of fibrinogen degradation products [31], changes in viscoelastic properties of fibrinogen [32,33,34], fibrinogen adsorption on surfaces [30,35,36] or generation of anticoagulant surfaces [37]. Rarely does the focus lie on the quantitative assessment of fibrinogen [25,38,39] like in this work, and if so, then the calculation of the fibrinogen concentration was usually based on aPTT measurements [25,38] instead of the more accurate Clauss assay [39]. In other publications complex, expensive and time-consuming quartz surface coatings were usually applied [25,38,39], mostly commercially available reference plasma was used [25,38] instead of the more challenging non-referenced biological assay mixture from healthy blood donors [39] and no comparison to multiple external reference methods was conducted.

We have successfully developed an advanced QCM-D sensor device for coagulation measurements [10] with a high degree of correlation to aPTT measurements [22,25]. The intention of the described approach here was to broaden the application spectrum of the device with the development of a QCM-D based detection method for simple, time- and cost-saving detection of fibrinogen levels in medical acute and routine testing for a clinical and non-clinical setting and to augment research on QCM-D-based quantitative fibrinogen assessment. In detail, the objective of the work was to

(1) transfer the CFA qualitatively to the QCM-D method and develop an easy, time- and cost-effective procedure (Section 3.1 and Section 3.2);
(2) transfer the CFA quantitatively to the QCM-D method: prepare calibration curves from reference pool plasma for three different QCM-D parameters (frequency signal shift (fQCM), calculated turning point of frequency signal shift (tfQCM) and dissipation signal shift (dQCM)) (Section 3.3); and
(3) conduct quantitative measurements with healthy donor’s plasma and compare the results to internal and external references (Section 3.4).

In contrast to the publications mentioned above which focus on QCM-D-based fibrinogen assessment, the presented work encompasses the following aspects:

- both qualitative and quantitative determination of fibrinogen with a QCM-D sensor;
- distinction of coagulation (plasma + thrombin) and blank sample (plasma + imidazole buffer; no coagulation induced) measurements in both frequency and dissipation signal;
- calculation of the fibrinogen concentration based on the Clauss fibrinogen assay instead of aPTT-based fibrinogen measurements;
- novel sensor surface coating approach: non-complex, inexpensive and prompt quartz surface coating is applied with donor own plasma;
- purchasable reference plasma is tested as well as the more challenging non-referenced biological assay mixture from healthy blood donors (real samples from healthy donors);
- three different QCM parameters (fQCM, frequency signal shift; dQCM, dissipation signal shift; and tfQCM, mathematically calculated turning point of fQCM) were considered;
- QCM-D results of CFA were compared to CFA results of internal and external reference methods (immunological assessment of fibrinogen, optical coagulometry, mechanical coagulometry).

## 2. Experimental Section

### 2.1. Blood Collection and Preparation

Fresh human whole blood was collected from healthy donors in 5 mL citrate tubes S-Monovette (Sarstedt, Nümbrecht—Rommelsdorf, Germany) and was centrifuged for 15 min (at 2500 g and 20 °C) for obtainment of platelet poor plasma (PPP). Platelet concentration of PPP was verified with blood count analyser CELL-DYN Ruby (Abbott Diagnostics, Lake Forest, IL, USA). Blood collection was approved by local ethics committee of University Hospital Tuebingen, Germany and performed by Institute for Clinical and Experimental Transfusion Medicine of University Hospital Tuebingen, Germany. Dilution of PPP was carried out with imidazole buffer (pH 7.35) that was made from imidazole (Trinity Biotech, Wicklow, Ireland) and sodium chloride (Sigma Aldrich, Taufkirchen, Germany). No identifying information on participant’s blood samples was used or is published. No animals were involved in the study.

### 2.2. QCM-D Sensor

For measurement, QCM-D was employed as transducer consisting of a piezoelectric 10 MHz AT-cut quartz sensor (8 mm in diameter, 166 µm thick) with two differently sized gold electrodes (upper side 8 mm, lower side 5 mm in diameter). Quartz surface cleaning was conducted with acetone solution (Sigma, Deisenhofen, Germany) for 1 min, rinsing with deionised water (laboratory supply) and blowing dry with nitrogen stream (built in laboratory supply). Blank gold (AU) quartz was used during measurements. No further sensor coating was used except for coating with donor own diluted plasma in certain experiments. Sensor was placed on a thin plastic carrier for sensor platform fitting.

### 2.3. QCM-D Sensor Platform and Signal Monitoring/Recording Software

The semi-automated sensor platform contains a thermostatic incubation block and is set at a constant temperature at 37 °C (non-commercially available forerunner model of the now commercially available qCell T, 3T analytik GmbH, Tuttlingen, Germany). The sensor platform includes one measurement chamber (capacity approx. 30 µL each) where the QCM-D sensor fixated on a plastic carrier is placed. The chamber is sealed with a lid module that consists of different microfluidic chips with three miniature tubings. With this setup the different liquids of the assay are metered fully automated, then mixed thoroughly in an Eppendorf cup and subsequently the mixture is injected into the measurement chamber. This is realised by PC-script automated pump that is integrated in the QCM-D device. If required, the sensor surface can be incubated with donor’s own PPP via pump automated by PC-script (=sensor surface incubation) before the mixture of the liquids is injected. After injection of the PPP and the assay mixture to the sensor surface, the pump stops in each case.

Signal changes of QCM-D were monitored and recorded on a computer with a software developed in our own research group. The dissipation signal (∆Γ values) presented is not calibrated to absolute values as the newly developed sensor platform was still in the development phase. Baselines of QCM-D signal changes are shifted in all following figures.

### 2.4. Merlin Coagulometer (Internal Reference), Centrifuge And Statistical Evaluation Software

Mechanical coagulometer Merlin MC 4 (Merlin Medical, ABW Medizin und Technik, Lemgo, Germany), Centrifuge Multifuge 3S-R Heraeus (VWR International GmbH, Darmstadt, Germany) and OriginPro 7.5 software (Origin Lab, Northampton, MA, USA) were used.

### 2.5. Reagents and Chemicals

Calibration Plasma “Coagulation Reference” consisting of pooled platelet poor citrate plasma from 100 healthy donors (Technoclone GmbH, Wien, Austria) was used and thrombin reagent “Fibrinogen reagent” containing 80 IU/mL bovine thrombin (Technoclone GmbH, Wien, Austria). According to manufacturer’s directions, lyophilised reagent was reconstituted with 5 mL distilled water, stored in refrigerator and incubated at room temperature before application. Physiological imidazole buffer (pH 7.35) for dilution of PPP was prepared (imidazole purchased from Trinity Biotech, Wicklow, Ireland and sodium chloride purchased from Sigma Aldrich, Taufkirchen, Germany). Hellmanex^®^ solution and Bandelin Sonorex^®^ ultrasound device (Bandelin electronic Berlin, Germany) for cleaning of sensor platform and lid modules were applied.

### 2.6. Experimental Procedure

#### 2.6.1. QCM-D Sensor Insertion and Start of Automated PC Script

**Figure 1:**
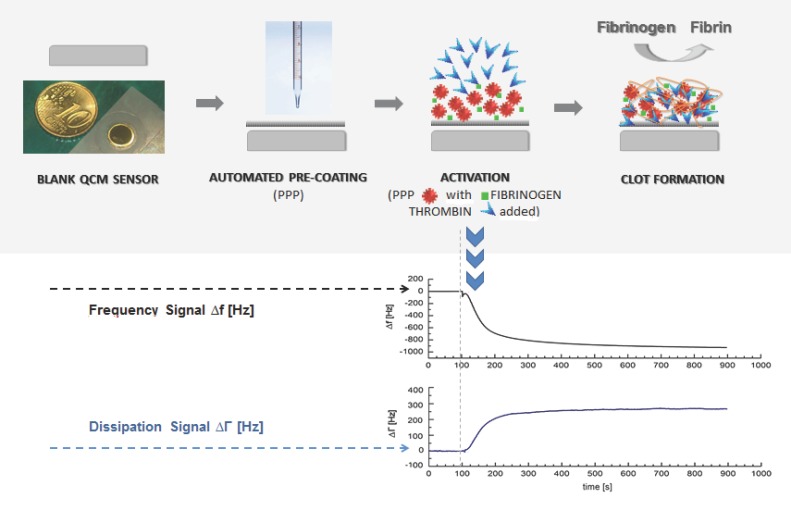

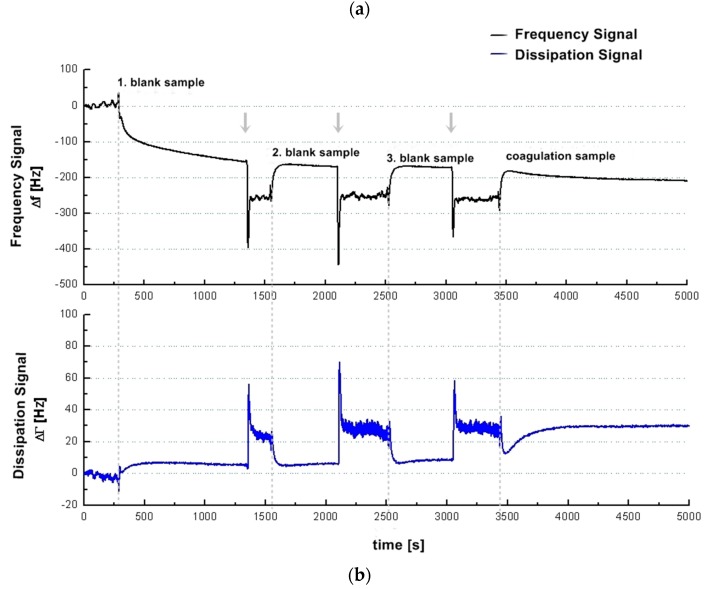

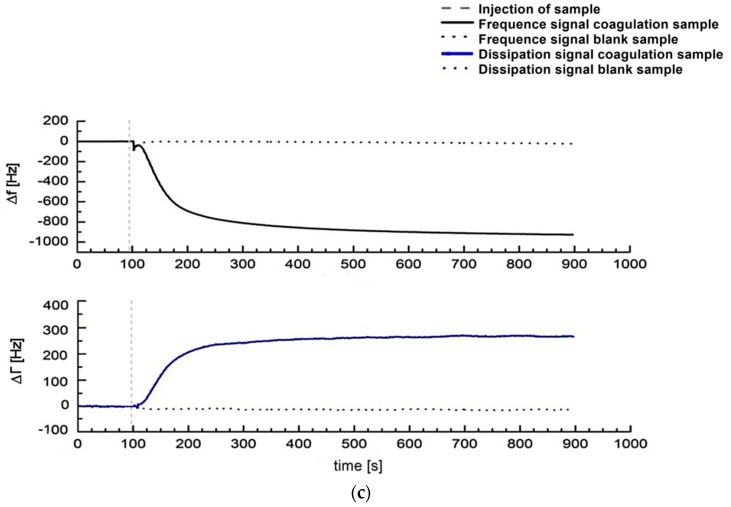
(**a**) Illustration of the general idea of a QCM-based coagulation measurement based on the Clauss assay on a pre-coated quartz (PPP); (**b**) Experimental series with consecutive injection of three blank samples (PPP + imidazole buffer, 1:10 diluted) and one coagulation sample (PPP + thrombin reagent, 1:10 diluted) from healthy donor I into the measurement chamber using healthy donor’s plasma and a blank quartz surface cleaned in acetone. Dotted vertical lines: script-automated injection of the mixed samples to the quartz chamber by pump that lasted several seconds. Then the pump was stopped. Arrows: new start of pump with imidazole buffer flow after each measurement in order to proceed with the experimental series until the injection of the next mixed sample; (**c**) 1:10 diluted measurements with coagulation sample (solid line, PPP + thrombin reagent) and blank sample (dotted line, PPP + imidazole buffer) of healthy donor II using a pre-coated quartz surface with 1:10 diluted donor’s own PPP. Vertical line: script-automated injection of the mixed samples to the quartz chamber by pump that lasted several seconds. Then the pump was stopped.

A plastic carrier with a QCM-D sensor was inserted into the measurement chamber of the sensor platform. Monitoring and recording was started. The measurement chamber was sealed with a lid module that consists of three different miniature tubings, and the following subsequent PC-script-automated steps that were carried out by the QCM-D device (also see Figure 1a):

(1) Optional: sensor surface coating with donor’s PPP (= sensor surface incubation), injection by QCM-D device

This step is optional in the automated script. Coating of sensor surface was automatically carried out by computer-based script. For this, 80 µL of donors’ 1:10 diluted PPP stored at 37 °C in an Eppendorf cup was applied to sensor surface with a pump that stopped afterwards. Approximately 120 s later the start of coagulation/blank sample measurement (see below) took place.

(2) Application of thrombin (blank samples: imidazole buffer) into adjacent Eppendorf tube filled with PPP by QCM-D device

Eppendorf tubes were each filled with PPP, imidazole buffer or thrombin reagent. PPP tubes were placed in platform’s incubation block (37 °C) shortly before measurement. For coagulation measurement, 180 µL thrombin reagent at room temperature was automatically added with pressure (generated by pump) to 180 µL of donor’s 1:10 diluted PPP at 37 °C by computer-based script. For blank sample measurements 180 µL imidazole buffer at room temperature was automatically added to 180 µL of donor’s 1:10 diluted PPP at 37 °C by computer-based script. Procedures were realised with equal mixture ratios.

(3) Injection of PPP—thrombin (blank sample: PPP—imidazole buffer) mixture onto quartz surface in measurement chamber by QCM-D device

This mixture (PPP + thrombin reagent or PPP + imidazole buffer) was immediately injected automatically by the device into the platform’s measurement chamber by pump via one of the tubings. Then the pump stopped.

#### 2.6.2. Generation of Calibration Curves

According to manufacturer’s directions, the calibration curve for Merlin coagulation time was developed with calibration plasma and thrombin reagent. 1 mL lyophilised citrate plasma was reconstituted with 1 mL of distilled water. Approximate value of fibrinogen content was 2.9 g/L. Different dilutions of calibration plasma (1:5, 1:10, 1:20, 1:40) were prepared with imidazole buffer and therefore contained different concentrations of fibrinogen (0.59 g/L, 0.29 g/L, 0.15 g/L, 0.07 g/L) based on the dilutions of the CFA calibration curves. Several measurements of each sample were carried out with the reference coagulometer and the QCM-D sensor platform, respectively. Results were recorded in a diagram and a linear fit was applied to the results with OriginPro 7.5 software.

### 2.7. Reference Coagulation Measurements

#### 2.7.1. Merlin Coagulometer (Internal Reference, MC)

The Merlin coagulometer assay is a mechanical (magnetic detection) end-point assay for fibrinogen measurement. Thrombin reagent Fibrinogen Reagent (Technoclone GmbH, Wien, Austria) was added manually to rotating plastic cuvette with PPP simultaneously to the start of QCM-D-based measurement. A rotating cuvette is inclined 6° to a thermostatic block (37 °C) and holds a stainless steel ball that is this way kept in position. Increase in blood viscosity forces the steel ball at a certain point to leave its position. This action is detected by magnetic field sensor and is used as coagulation time. The procedure was realised with equal mixture ratios and volumes as the QCM-D procedure. The fibrinogen concentration was calculated by coagulation time on the basis of the calibration curve established from reference pool plasma (Technoclone GmbH, Wien, Austria) according to manufacturer’s directions.

#### 2.7.2. Turbidimetric Fibrinogen Assay, Central Laboratory of University Hospital of Tuebingen, Germany (External Reference, UKT-ZL)

A turbidimetric (photo-optical detection) assay for fibrinogen measurement was conducted. Photo-optical systems depend on a change in optical density resulting from fibrin formation. Thrombin reagent HemosIL Fibrinogen C XL (Instrumentation Laboratory, Munich, Germany) was added fully automated by ACL TOP device (Instrumentation Laboratory, Munich, Germany) to PPP diluted with factor diluent (Instrumentation Laboratory, Munich, Germany) in a ratio of 1:10. The fibrinogen concentration was calculated based on calibration curve established from the reference pool plasma HemosIL-Calibration Plasma (Instrumentation Laboratories, Munich, Germany) according to manufacturer’s directions.

#### 2.7.3. Turbidimetric Fibrinogen Assay, Coagulation Laboratory of University Hospital of Tuebingen, Germany (External Reference, UKT-GL)

A turbidimetric (photo-optical detection) assay for fibrinogen measurement was conducted. Thrombin reagent Multifibren*U (Siemens, Marburg, Germany) was added fully automated by a Behring Coagulation Timer (BCT) device (Siemens, Marburg, Germany) to PPP diluted with imidazole buffer solution (Siemens, Marburg, Germany) in a ratio of 1:10. The fibrinogen concentration was calculated based on the calibration curve established from reference pool plasma Kalibrator Kit (Siemens, Marburg, Germany) according to manufacturer’s directions.

#### 2.7.4. Rapid Immunodiffusion Fibrinogen Assay, Coagulation Laboratory of University Hospital of Tuebingen, Germany (External Reference, UKT-IM)

A rapid Immunodiffusion assay for fibrinogen measurement was conducted. Employed was the agar plate NOR-Partigen^®^ Fibrinogen (Siemens, Marburg, Germany) with 12 gel wells and control of success N/T Protein Kontrolle PY (Siemens, Marburg, Germany). The interpretation took place using the MACINI method after 48 h storage at room temperature and was based on the provided benchmark table according to manufacturer’s directions.

## 3. Results and Discussion

### 3.1. Qualitative Transfer of the Clauss Fibrinogen Assay to the QCM-D method—QCM-D-Based Determination of Fibrinogen in Healthy Human Donors: Signal Characteristics and the Significance of the Sensor Surface Incubation with Donor Own Plasma

In order to verify that the CFA can be qualitatively adapted to the QCM-D, the QCM-D signal characteristics of the assay had to be elaborated. The behaviour of the QCM-D signals during CFA coagulation measurements (PPP + thrombin reagent) and blank samples (PPP + imidazole buffer) as well as the meaning of sensor surface coating are shown in Figure 1.

Figure 1a illustrates the general idea of the presented coagulation measurements. Figure 1b shows an experimental series with consecutive injection of three blank samples (PPP + imidazole buffer) and one coagulation sample (PPP + thrombin reagent) from donor I. The first blank sample was injected onto a blank quartz surface cleaned in acetone. The second blank sample was injected onto the same quartz sensor. Therefore, the 2nd, 3rd and 4th injections correspond to a measurement with pre-treatment of quartz surface with donor’s own PPP. The quartz sensor was left in the measurement chamber between different injections. Figure 1c shows the signal change in the frequency and dissipation signal comparing a coagulation sample (solid line, PPP + thrombin reagent) and a blank sample (dotted line, PPP + imidazole buffer) of donor II using a pre-coating on the quartz surface with 1:10 donor’s PPP. The behaviour of the frequency and dissipation signal before injection of the mix onto the quartz surface by pump is in both cases identical, and the solid line covers the dotted line in the graph. Figure 1b (coagulation sample) and Figure 1c demonstrate that the QCM-D-based CFA results in frequency and dissipation signal shifts. Based on the way QCM-D sensors operate (signal shifts due to changes in mass, viscosity and viscoelasticity) this is the signal reaction expected. Therefore fibrinogen can qualitatively be detected with a QCM-D sensor.

One of the problems we were facing with the QCM-D-based CFA was the non-coagulation-dependent signal change (see Figure 1b, 1st blank sample) in the frequency signal. Using an uncoated gold sensor in order to keep the preparation process as brief and the material costs as low as possible, coagulation samples cannot be clearly distinguished from blank samples. We refer this strong signal change (Figure 1b, 1st blank sample) to a non-specific plasma protein coupling on the uncoated gold quartz surface after sample injection as the occurrence of coagulation is always related to a deposition of a viscoelastic layer and results in a change of dissipation. For ideal sensor coating conditions only the coagulation process should contribute to the signal. Therefore the unspecific protein coupling has to be eliminated. Figure 1b,c show that using the surface coating with 1:10 diluted PPP the unspecific protein coupling can be eliminated.

Compared to other surface coatings used for QCM-D-based coagulation measurements (*i.e.*, polystyrene [38,39] or polyethylene [40]) the donor’s own PPP surface coating is interesting because

- the use of donor’s own plasma is inexpensive: no costs for expensive coating material;
- the use of donor’s own plasma is conducted quickly: no time spent for coating and no time needed for coating to dry, coating with donor’s own plasma can be automatically conducted approximately 120 s before the coagulation measurement; and
- there is no interdependency with extraneous material.

The pre-treatment with 1:10 diluted plasma proved especially convenient, since

- the standard Clauss assay is also executed with a dilution of 1:10;
- the coating material can be drawn from the 1:10 diluted plasma to be used later for measurements (fast coating procedure and compatible process); and
- injections to the measurement chamber are operated automatically by PC-script that manages injections for pre-treatment and regular measurement.

In comparison to the qualitative signal results of QCM-D-based fibrinogen measurements presented by Yao, Qu and Fu [39] we do have very similar frequency signal curves measuring blank and coagulation samples. Their signal change at the start point after activation (called start-point (t_1_) in their paper) looks alike. Nevertheless, in our frequency signal diagrams there is no additional signal detectable within 5 min (referred to as end-point (t_2_) of coagulation in their paper). A possible explanation for this can be the influence of the different quartz surfaces (polystyrene *vs.* gold and PPP) for the measurements and the determination of aPTT-related fibrinogen levels instead of the CFA.

### 3.2. Qualitative Transfer of the Clauss Fibrinogen Assay to the QCM-D Method—QCM-D-Based Determination of Fibrinogen from Healthy Human Donors: QCM-D Signal Changes during Coagulation—Effects of Mass, Viscosity or a Combination of Both? Evaluation by Means of ∆f vs. ∆Γ Diagrams

**Figure 2:**
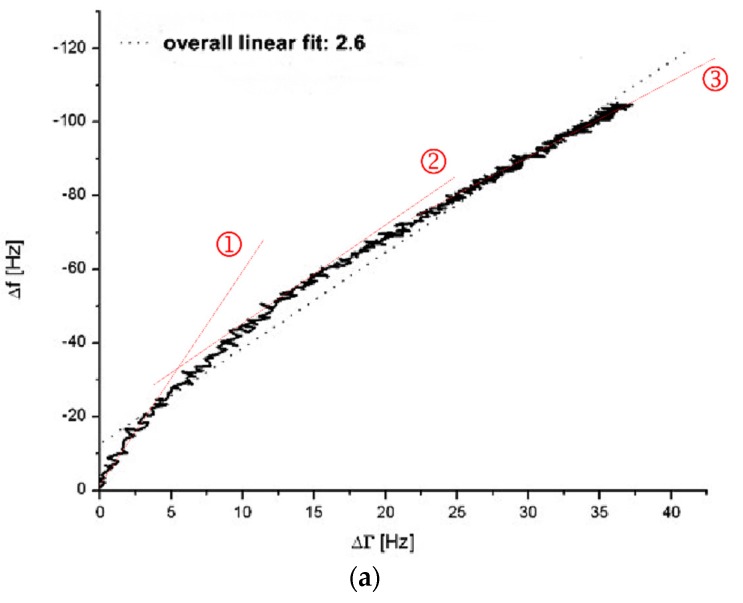

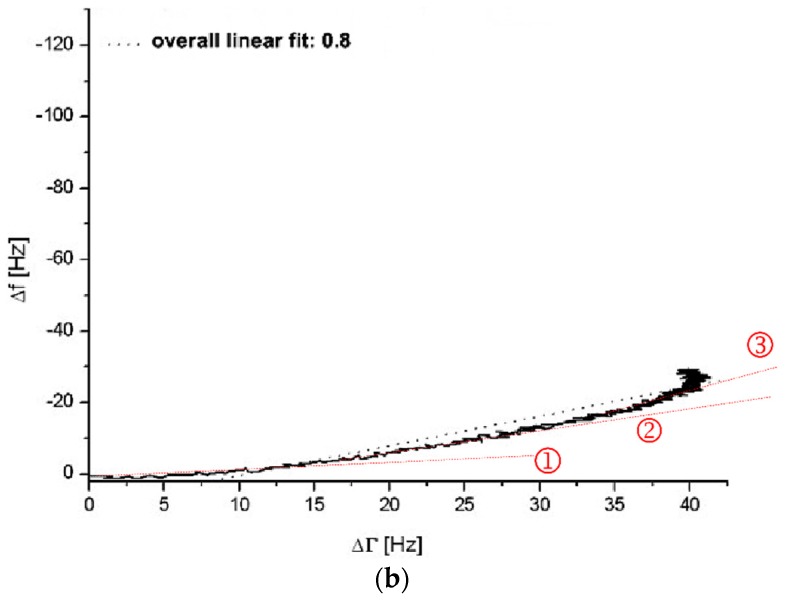
∆f *vs.* ∆Γ diagrams without (**a**) and with (**b**) incubation of the quartz surface with 1:10 diluted healthy donor’s PPP (donor III). In figure (**a**) and (**b**) a coagulation measurement (PPP + thrombin reagent, 1:10 diluted) was run for 900 s. Grey dotted lines show overall linear fit. Red dotted lines marked with ①, ② and ③ show different linear fits during different stages of the coagulation process.

With future quantitative fibrinogen measurements in mind, it was important to find out whether the observed signal changes were caused by mass adsorption only, by changes in viscosity or by a combination of both. Mass effects are effects based on the change of the mass that is coupled to the sensor surface. Viscosity changes are effects based on the changes of the viscosity during the coagulation process of the injected assay mixture. However, mass effects can occur due to deposition of proteins, fibrinogen-/fibrin strands or the weight of the whole coagulation clot on the quartz surface. The underlying causes of signal changes can be distinguished using the ∆f *vs.* ∆Γ diagram [10]. In the ∆f *vs.* ∆Γ diagram, the frequency signal changes ∆f are shown on the y-axis whereas the corresponding dissipation signal changes ∆Γ are shown on x-axis. Then the curve gradient (overall linear fit) is calculated by linear fit function. When recording frequency and dissipation signal changes in a ∆f *vs.* ∆Γ diagram, pure viscosity triggered changes result in a line through origin with gradient 1 [10] as ∆f = ∆Γ for viscous Newtonian liquids like PPP [41]. If ∆f > ∆Γ the gradient is >1 which stands for signal changes due to mass effects. Figure 2 shows the ∆f *vs.* ∆Γ diagram of a coagulation measurement performed on a blank (Figure 2a) and donor’s own PPP pre-treated (Figure 2b) quartz sensor.

The coagulation measurement with the non-pre-treated gold surface presents with an overall linear fit gradient of 2.6 (see grey dotted line Figure 2a) whereas with the pre-treated surface presents with a gradient of 0.8 (see grey dotted line Figure 2b). Even though there is a deviation of the presented curves in Figure 2 from accurate linear behaviour, the overall linear fit gradient gives an idea of the quartz’s behaviour conducting a QCM-D-based CFA. The overall linear fit gradient of 2.6 suggests that the signal changes are rather due to changes in surface coupled mass than due to changes in viscosity. With an overall linear fit gradient of 0.8 the measurement on the pre-treated quartz sensor is close to 1. Therefore it indicates almost pure signal changes based on changes in viscosity. The results from Figure 2 verify the findings in Figure 1 and confirm the arguments of protein-based mass adsorption on the sensor surface that lead to strong frequency signal changes if the surface is not blocked with PPP.

Closer examination of the ∆f *vs.* ∆Γ diagrams gives an interesting insight into the behaviour of the coagulation process on the QCM-D. The gradients change throughout the measurement (compare red dotted lines (slopes) at stages ①, ② and ③ in Figure 2a,b. This explains to a high degree the deviation of the overall linear fit. On an uncoated quartz (Figure 2a) the curve begins with a very steep slope (Figure 2a(①)), representing massive adsorption which gets less steep over time (Figure 2a(②,③)) as the surface saturates. The initial massive adsorption on uncoated quartzes is undesired since it handicaps the distinction between blank and coagulation samples when it comes to quantitative measurements of the fibrinogen levels. On a coated quartz (Figure 2b) the slope kinetic is inverse to an uncoated quartz (Figure 2a) with virtually no mass effect in the beginning (Figure 2b(①)) and mostly changes in viscosity. Interestingly, over time the mass effect increases (Figure 2b(②,③)) due the coupling of the clot and the fact that the fibrin net renders the viscoelastic coating more rigid.

Overall, for the uncoated quartzes (Figure 2a) and the last sequence of the PPP coated quartzes (Figure 2b(③)), ∆f values change more than those of ∆Γ. Therefore, mass effects still contribute to the presented QCM-D results—even with the blocking of the sensor surface by plasma incubation. This is a situation (∆f > ∆Γ) which Lakshmanan *et al.* identified likewise during their QCM-D-based fibrinogen measurements, despite complex polystyrene surface coating [38]. The mass adsorption is not necessarily only due to random protein adsorption on the sensor surface. We believe that the mass effect using a PPP-coated quartz is more likely caused by

- the cumulative growth and weight of the forming coagulation clot;
- the attachment of the forming coagulation clot to the sensor surface that is facilitated by binding to the donor’s own PPP layer;
- the change from soluble fibrinogen to insoluble fibrin; or
- a combination of the different aspects.

### 3.3. Quantitative Transfer of the Claus Fibrinogen Assay to the QCM-D Method—Generation of Serial Dilutions and Calibration Curves for Three Different QCM-D Parameters (fQCM, dQCM, tfQCM) from Reference Pool Plasma

**Figure 3:**
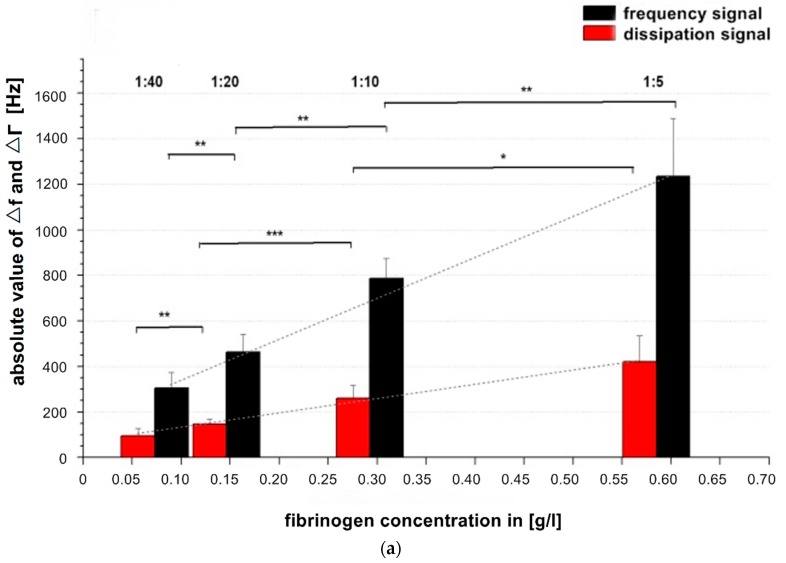

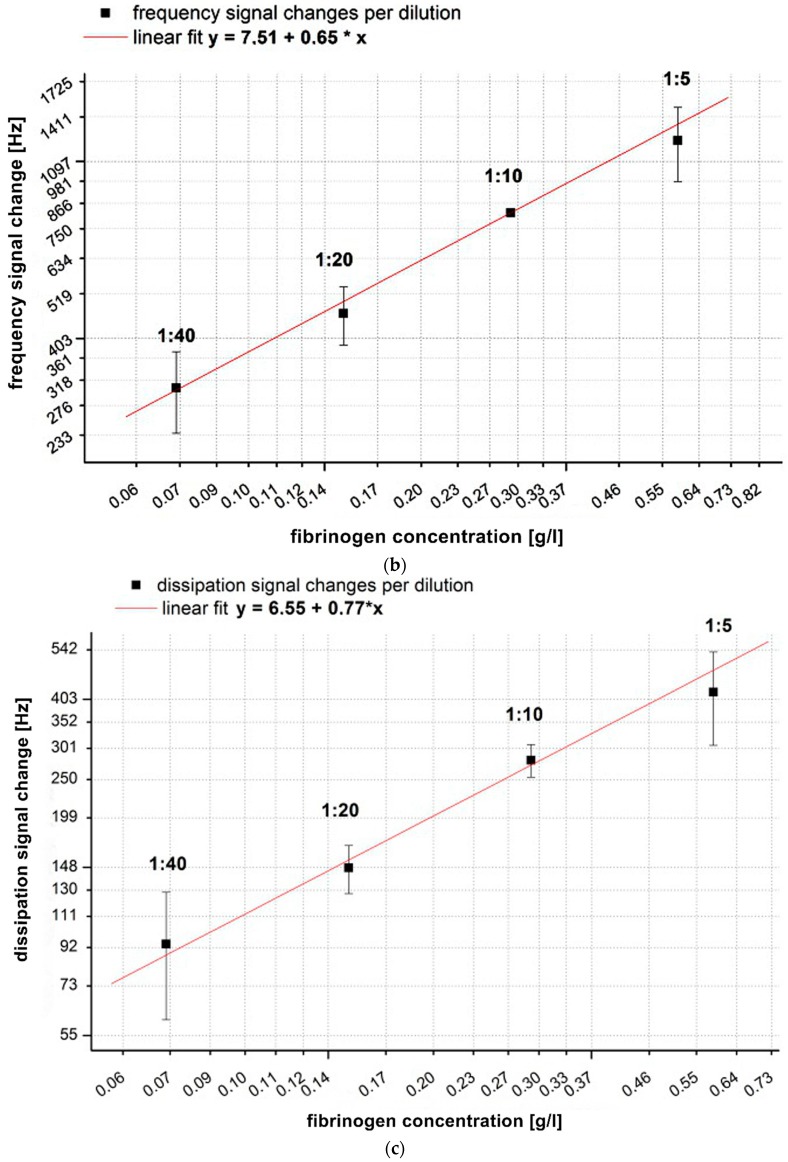

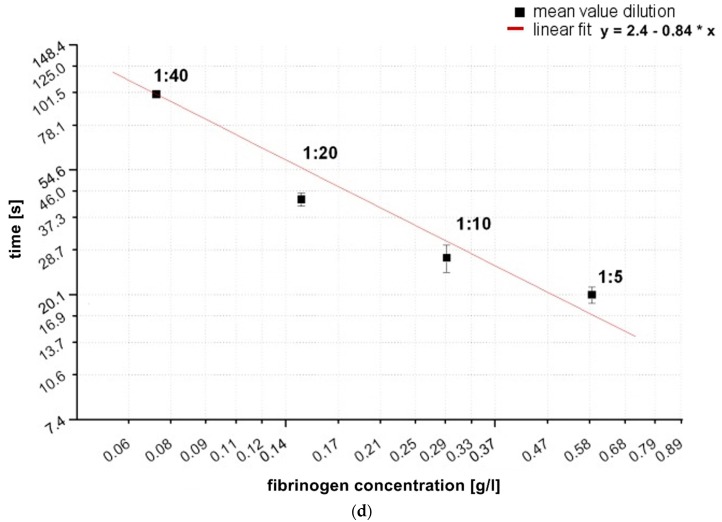
(**a**) Comparison of absolute values of frequency and dissipation changes shown on a double logarithmic scale with reference pool plasma dilutions of 1:5, 1:10, 1:20, 1:40. All quartzes were pre-coated with the correspondent dilution of the calibration measurement. The QCM-D signal changes were determined at t = 900 s after starting coagulation by adding thrombin. n = 6 measurements per dilution. Shapiro-Wilk-Test for testing of normal distribution and ANOVA for variance analysis. Significance is indicated with asterisks as follows: * = significant with *p* ≤ 0.05, ** = very significant with *p* ≤ 0.01, *** = highly significant with *p* ≤ 0.001. Grey dotted line indicates the different slopes s of linear fit curves of the dissipation (s = 789) and frequency signal (s = 2284) concerning signal change per g/L; (**b**) Generated calibration curve of fQCM by different dilutions; (**c**) Generated calibration curve of dQCM by different dilutions; (**d**) Generated calibration curve of Merlin coagulometer by different dilutions. Logarithmic *x*- and *y*-axis display of fibrinogen concentration and signal changes in (**b**–**d**); red lines illustrate linear fit of mean values per dilution.

In order to evaluate the QCM-D-based CFA in regard to possible quantification of fibrinogen in plasma, we applied a protocol analogue to the establishment of CFA calibration curves. A dilution series of reference pool plasma ranging from 1:5 to 1:40 (corresponding to a range of 0.59 g/L to 0.07 g/L fibrinogen) was measured. ∆f (Figure 3a,b), ∆Γ (Figure 3a,c) were determined and the turning point of the frequency signal shift (tfQCM) (Figure 3d) was calculated from the ∆f curve. tfQCM is defined as the time difference ∆t between thrombin injection and the mathematically calculated turning point of the frequency curve (OriginLab software). Using a 1:10 dilution, the tfQCM values matched with the Merlin-based coagulation values.

In Figure 3a all signal changes per concentration are significant. As the concentration of fibrinogen increases, the value of ∆f also increases, indicating a larger fraction of mass attached to the surface. At the same time the value of ∆Γ increases, indicating increased viscosity. Our results are in agreement with and confirm the results of Lakshmanan *et al.* [38,42] and Hussain *et al.* [25] by showing that signal changes depend on the fibrinogen concentration and that ∆f changes more with raising fibrinogen concentration than ∆Γ ( see grey dotted slope lines Figure 3a).

### 3.4. Quantitative Transfer of the Clauss Fibrinogen Assay to the QCM-D Method: Measurement of Unknown Fibrinogen Concentration in Six Healthy Donor’s Plasma with the Use of the Established Calibration Curves and Comparison of the Results to Common References

**Figure 4:**
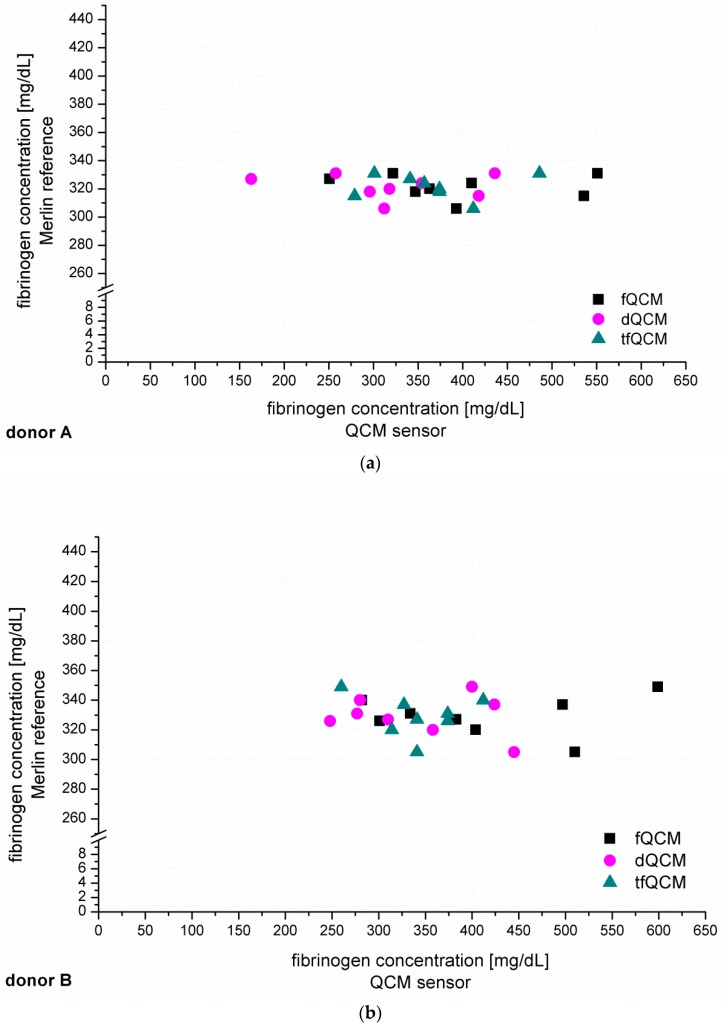

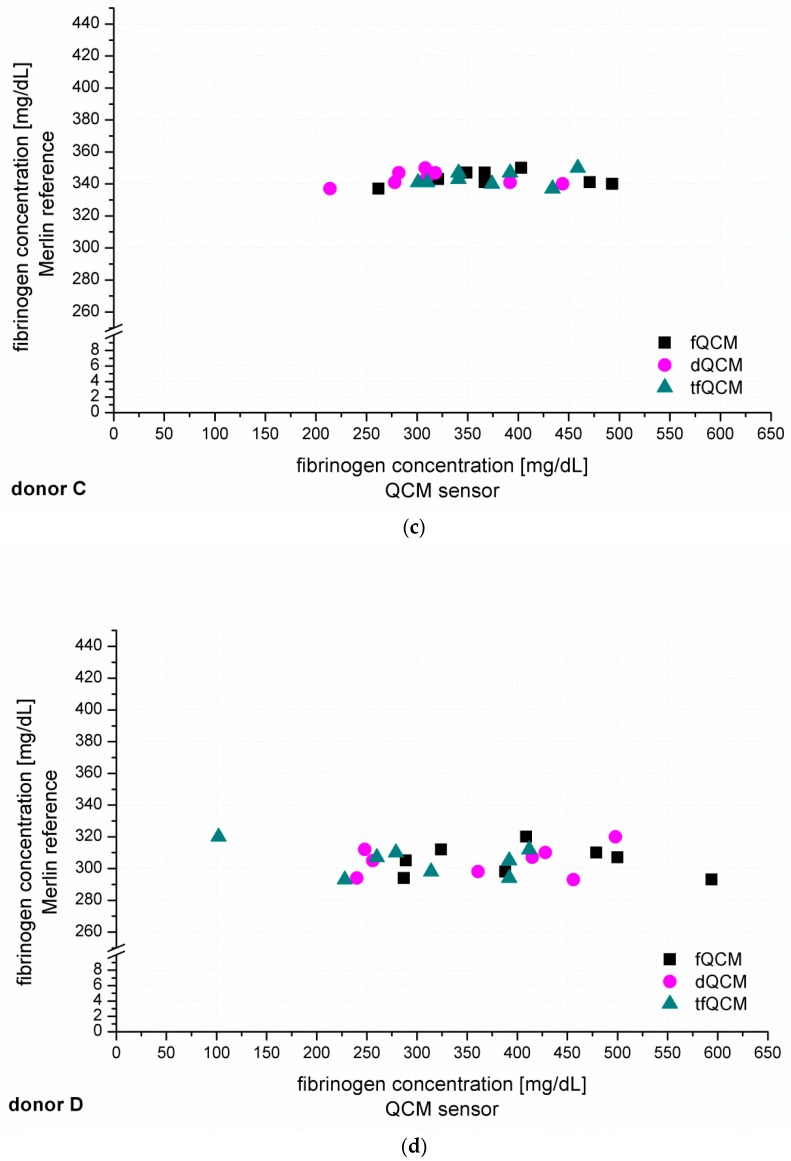

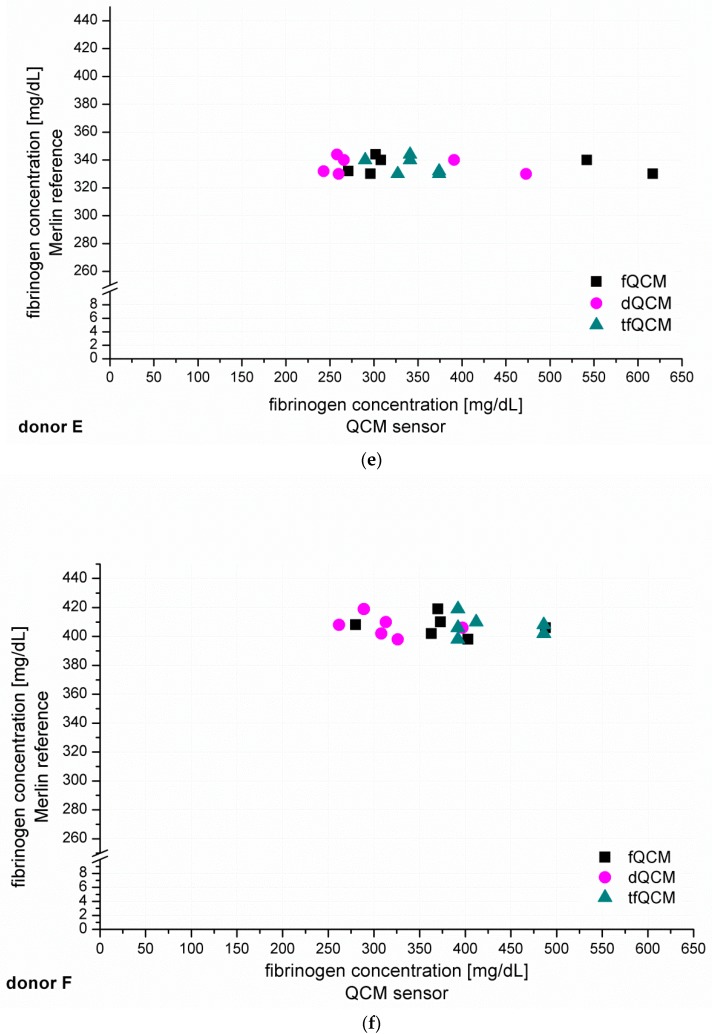
(**a**–**f**) show the individual results of the QCM-D-based determination of unknown fibrinogen levels conducted for six healthy blood donors (donor A–F). The QCM-D measurements were conducted n = 8 times for donor A–D and n = 6 times for donor E–F. The experiments were conducted with 1:10 diluted plasma and accordingly coated quartzes. The y-coordinate shows the results of the simultaneously conducted internal Merlin reference whereas the x-coordinate shows the QCM-D results in direct comparison.

After the establishment of calibration curves, the next step was to identify unknown fibrinogen concentrations in PPP from six healthy donors (donor A–F, Figure 4). The recorded QCM-D signals for fQCM (■), dQCM (●) and tfQCM (▲) were evaluated and fibrinogen concentrations calculated by comparison with the calibration curves. Simultaneous measurements with the Merlin coagulometer served as an internal reference using identical plasma specimen and coagulation reagents.

Figure 4 shows that fQCM (■)-based fibrinogen values tend to indicate higher fibrinogen concentrations than the dQCM (●) and tfQCM (▲)-based values do. tfQCM (▲) values are mainly close together whereas fQCM (■) and dQCM (●) values tend to spread broader. The considerable scattering of the different QCM-D results per donor shows that the accordance of individual QCM-D-based CFA measurements has to be further optimized. At this point an individual QCM-D-based CFA measurement could not yet be used for dependable results in medical care. Possible factors influencing the deviation of the single QCM-D measurements are:

- duration of measurement series per donor lasted > 3 h what can lead to deviation of results [43];
- age, storage life, stability and service temperature of thrombin reagent during long lasting measurements;
- the microfluidic sample delivery modules for this application were specially manufactured prototypes and may have potential for further standardization;
- points 1–3 combine and add up during the CFA measurements; and
- points 1–3 already combined and added up throughout the generation of the calibration curves—due to the error propagation the deviation is increased.

In addition to the individual QCM-D results of donor A–F presented in Figure 4 we averaged the individual QCM-D results per donor in order to calculate the mean value of the fibrinogen concentration results of donor A–F. The numeric results of the fibrinogen concentration obtained that way for fQCM, dQCM and tfQCM are summarized in Table 1, lines 5, 7 and 9. The corresponding Merlin-based fibrinogen levels that served as an internal reference are listed in line 4. The corresponding fibrinogen levels received from standardized external references (UKT-ZL, UKT-GL, UKT-IM) are displayed in lines 1–3.

**Figure 5:**
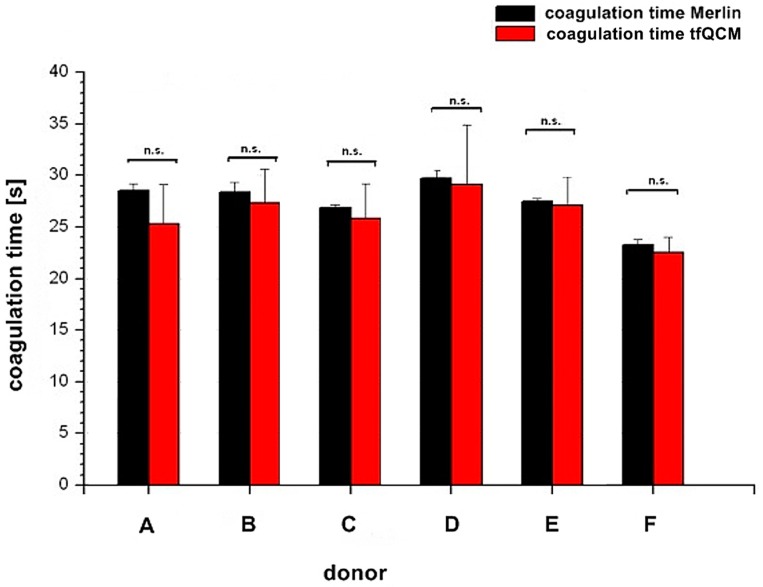
Comparison of coagulation times between Merlin coagulometer (black column) and tfQCM (red column) based on the fibrinogen concentration results in Table 1. The x-axis shows the different donors A–F. The y-axis shows the coagulation time. Results of coagulation time from both parameters are not significant (n.s.) with *p* ≥ 0.05.

Figure 5 illustrates the comparison of the coagulation time measured with the internal Merlin reference and the tfQCM that led to the fibrinogen concentration results of donor A–F in Table 1, lines 4 and 5.

As expected, the results of the external references in Table 1 do correspond very well with each other. The QCM-D-based results present higher fibrinogen values than the external reference methods for donor A–F. The internally standardized Merlin reference does not correspond well with the results of the externally standardized references. It rather matches with the higher QCM-D-based results. This effect can probably be attributed to the fact that the Merlin measurements were conducted at the same time, with identically treated plasma and an identical thrombin reagent. The thrombin reagent used for the QCM-D and Merlin-based results was compared to the one used at the external central laboratory of the university hospital (UKT-ZL) by the reference institute of bioanalytics in Bonn, Germany, sending blind samples for testing to laboratories all over Germany. The comparison demonstrated that the reagent used by the UKT-ZL constantly resulted in slightly lower fibrinogen results [44]. Most likely, that finding does not solely explain the presented differences in results, but it definitely contributes a substantial part. Studies show that fibrinogen results vary widely depending on

- incorrect calibration of the commercially available reference plasma [1,45,46,47];
- varying thrombin concentration of 35–200 U/mL in commercially available thrombin reagents [1];
- use of different calibrators for generation of calibration curves [48]; and
- use of different thrombin reagents [48].

The external reference results did only show agreement to the Merlin and QCM-D method with a continuous shift of about 82 mg/dL (SD ± 10.5 mg/dL) fibrinogen per donor (Table 2). Even though the Merlin method is known to produce slightly higher results than optical methods [45,49,50] it is again very unlikely for this to be the only explanation for the deviation. As there were no platelets in the PPP (tested with cell counter after blood centrifugation), this explanation can also be eliminated as a possible influence factor. Having all those different aspects in mind, probably a combination of the mentioned aspects should be considered when interpreting the presented results.

Whereas for several reasons there is no direct agreement of the QCM-D-based CFA with external references, the agreement of the standardized Merlin method and the new QCM-D method is good to excellent if using the mean values of the QCM-D measurements. Excellent agreement with the results of the standardized Merlin coagulometer is provided by tfQCM (see Table 1, lines 4 and 5 and Figure 5) and therefore shows the successful quantitative adaption of the CFA to the QCM-D method. Even though the standard deviation of tfQCM remains higher than the Merlin’s, the obtained results do not differ significantly, and with the exception of donor A the results of donor B–F lie within the aberration rate of 30 mg/dL proclaimed acceptable for clinically relevant decisions [45]. As the single tfQCM values scatter the least (Figure 4a–f) the averaged tfQCM-based fibrinogen determination shows a comparatively low standard deviation (Table 1, line 6).

The excellent results with tfQCM are followed by good results with dQCM (Table 1, line 7) and mostly good results with fQCM (Table 1, line 9). Compared to averaged tfQCM values, averaged fQCM and dQCM results differ more often from the Merlin results, do have higher standard deviations and often lie out of the limits for therapeutically relevant decisions (Table 1, line 7–10). fQCM and tfQCM results present—except from donor D—noticeably higher than dQCM results. That is consistent as tfQCM is calculated from fQCM data. The combination of the averaged results of all three QCM parameters per donor does not optimize the agreement of the QCM method with the reference methods.

In terms of time efficiency the excellent agreement of tfQCM with the results of the Merlin coagulometer is a great benefit for the QCM-D-based CFA as tfQCM can be obtained in less than 60 s after the injection of the assay mixture into the measurement chamber. In comparison, the fQCM and dQCM values in the presented experiments are obtained at t = 900 s after injection. The coagulation measurement in Figure 1c shows that due to a lack of significant signal changes, results could already be obtained at t = 400–500 s. Lakshmanan *et al.* [38] describe a similar time frame for results to be obtained for fQCM and dQCM after 500 s. Nevertheless there remains a great time discrepancy using fQCM or dQCM in comparison to tfQCM.

Yao *et al.* [39] detected a steep QCM frequency signal increase after the initial frequency change approximately three minutes after injection of thrombin reagent to plasma using a polystyrene coated quartz. In their paper the time between the injection and the frequency change matched the coagulation time of an optical coagulometer and a quantitative fibrinogen assessment could be conducted. A comparable frequency change after injection of thrombin was not found with the presented CFA measurement methods.

As fibrinogen concentration influences ∆f and ∆Γ, Lakshmanan *et al.* [38] presented a formula for the semi-quantitative evaluation of fibrinogen from aPTT measurements in commercially available reference plasma. According to them the formula can be used to determine fibrinogen concentration in a reference plasma sample from a calibration curve obtained from normal plasma controls. With the presented experimental setup and the use of plasma from healthy donors, the formula could not be successfully applied to the QCM-D technology presented in this work. Given that the formula of Lakshmanan *et al.* implicates the need to identify and evaluate two QCM parameters (frequency and dissipation signal) and that the results can only be obtained after 500 s, as well as considering the fact that the determination of fibrinogen by Clauss assay is to be favoured [1] we recommend the QCM-D-based Clauss assay approach with donor own PPP coating presented in this work for further research.

## 4. Conclusions/Outlook

The presented work demonstrates and verifies the principal suitability of QCM-D sensors for the qualitative and quantitative realization of the CFA, as different fibrinogen concentrations relevant for the CFA resulted in significant frequency and dissipation signal changes. In this work we showed that:

(1) the use of an inexpensive and time efficient surface coating with donor own PPP was successful;
(2) within the QCM-D parameter tfQCM, the QCM-D-based results of the CFA are available comparably promptly (<60 s);
(3) the determination of fibrinogen levels with a QCM-D sensor can be conducted in the Clauss assay setting (which is the gold standard);
(4) the results were drawn from the more challenging non-referenced biological plasma from healthy donors instead of commercial reference plasma;
(5) three different QCM-D parameters (fQCM, dQCM and tfQCM) were analysed; and
(6) the QCM-D results were compared to four reference methods with excellent agreement with the internal reference (same conditions) and a result shift to external references that can be explained by experimental and procedural setup.

Even though, for further validation, the sample number has to be raised and samples from a broader spectrum of pathological blood and blood containing anticoagulants or other drugs have to be tested in the near future, several challenges have been identified within this work which will allow technical and procedural adjustments to further optimize the method.

The presented QCM-D sensor method for the assessment of fibrinogen levels provides great advantages, including savings of cost and time and a great potential for miniaturization and point of care measurements. Since the surface coating is integrated as part of the measurement setting, quartzes can be stored easily and no interaction with external coating materials needs to be feared. The QCM-D device we used is small (260 × 317 × 197 mm^3^) and easy to handle due to its automated script controlled operation. There is no delay in transportation time, sample turn or result processing. The results draw attention to new and interesting alternatives for the assessment of fibrinogen levels by QCM-D sensors.

## Figures and Tables

**Table 1:** Numeric overview of the fibrinogen results of donor A–F based on the mean values of the QCM-D method, the internal reference (Merlin coagulometer) and the standard laboratory testing of three different standardized external references (UKT-ZL, UKT-GL, UKT-IM). The QCM-D-based numeric results are the calculated mean values of the measurements presented in Figure 4. The standard deviation (SD) between the QCM-D measurements is indicated.

Line	Method	Donor A (mg/dL)	Donor B (mg/dL)	Donor C (mg/dL)	Donor D (mg/dL)	Donor E (mg/dL)	Donor F (mg/dL)
**1**	**UKT-ZL (external reference)** Turbidimetric fibrinogen assay, Central Laboratory of University Hospital Tuebingen	**247**	**243**	**242**	**237**	**256**	**315**
**2**	**UKT-GL (external reference)** Turbidimetric fibrinogen assay, Coagulation laboratory of University Hospital Tuebingen	235	242	245	231	235	338
**3**	**UKT-IM (external reference)** Rapid immunodiffusion fibrinogen assay, coagulation laboratory of University Hospital Tuebingen	226	238	268	280	268	352
**4**	**Merlin (internal reference)** Mechanical fibrinogen assay	**322**	**329**	**343**	**305**	**336**	**407**
**5**	**Average tfQCM from ∆f**	**366**	**344**	**369**	**298**	**341**	**425**
**6**	***SD tfQCM***	*±65*	*±46*	*±57*	*±120*	*±32*	*±50*
**7**	**Average dQCM**	319	343	318	364	291	316
**8**	***SD dQCM***	*±87*	*±51*	*±71*	*±102*	*±108*	*±45*
**9**	**Average fQCM**	397	414	379	408	389	379
**10**	***SD fQCM***	*±103*	*±112*	*±76*	*±110*	*±150*	*±67*

**Table 2:** Numeric overview of shifts between fibrinogen results of laboratory internal Merlin coagulometer (Merlin) and the external central laboratory of the university hospital (UKT-ZL).

	Donor A (mg/dL)	Donor B (mg/dL)	Donor C (mg/dL)	Donor D (mg/dL)	Donor E (mg/dL)	Donor F (mg/dL)
**UKT-ZL (external reference)** Turbidimetric fibrinogen assay, Central Laboratory of University Hospital Tuebingen	247	243	242	237	256	315
**Merlin (internal reference)** Mechanical fibrinogen assay	322	329	343	305	336	407
**Difference**	**75**	**86**	**101**	**68**	**80**	**92**

## References

[1] Mackie I.J., Kitchen S., Machin S.J., Lowe G.D. (2003). Guidelines on fibrinogen assays. Br. J. Haematol..

[2] Brohi K., Cohen M.J., Davenport R.A. (2007). Acute coagulopathy of trauma: Mechanism, identification and effect. Curr. Opin. Crit. Care.

[3] Maegele M., Lefering R., Yucel N., Tjardes T., Rixen D., Paffrath T., Simanski C., Neugebauer E., Bouillon B. (2007). Early coagulopathy in multiple injury: An analysis from the german trauma registry on 8724 patients. Injury.

[4] Kamath S., Lip G.Y. (2003). Fibrinogen: Biochemistry, epidemiology and determinants. QJM.

[5] Canseco-Avila L.M., Jerjes-Sanchez C., Ortiz-Lopez R., Rojas-Martinez A., Guzman-Ramirez D. (2006). Fibrinogen. Cardiovascular risk factor or marker?. Arch. Cardiol. Mex..

[6] Koenig W. (2003). Fibrin(ogen) in cardiovascular disease: An update. Thromb. Haemost..

[7] Danesh J., Lewington S., Thompson S.G., Lowe G.D., Collins R., Kostis J.B., Wilson A.C., Folsom A.R., Wu K., Benderly M. (2005). Plasma fibrinogen level and the risk of major cardiovascular diseases and nonvascular mortality: An individual participant meta-analysis. JAMA.

[8] Sauerbrey G. (1959). Verwendung von schwingquarzen zur wägung dünner schichten und zur mikrowägung. Z. Phys. A Hadrons Nucl..

[9] Kanazawa K.K., Gordon J.G. (1985). Frequency of a quartz microbalance in contact with liquid. Anal. Chem..

[10] Gehring F.K. (2005). Schwingquarzsensorik in Flüssigkeiten—Entwicklung Eines Blutanalysegerätes.

[11] Johannsmann D. (2008). Viscoelastic, mechanical, and dielectric measurements on complex samples with the quartz crystal microbalance. Phys. Chem. Chem. Phys..

[12] Du B., Johannsmann D. (2004). Operation of the quartz crystal microbalance in liquids: Derivation of the elastic compliance of a film from the ratio of bandwidth shift and frequency shift. Langmuir.

[13] Bandey H.L., Martin S.J., Cernosek R.W., Hillman A.R. (1999). Modeling the responses of thickness-shear mode resonators under various loading conditions. Anal. Chem..

[14] Martin S.J., Granstaff V.E., Frye G.C. (1991). Characterization of a quartz crystal microbalance with simultaneous mass and liquid loading. Anal. Chem..

[15] Granstaff V.E., Martin S.J. (1994). Characterization of a thickness-shear mode quartz resonator with multiple nonpiezoelectric layers. J. Appl. Phys..

[16] Bandey H.L., Cernosek R.W., Lee W.E., Ondrovic L.E. (2004). Blood rheological characterization using the thickness-shear mode resonator. Biosens. Bioelectron..

[17] Efremov V., Killard A.J., Byrne B., Lakshmanan R.S. (2013). The modelling of blood coagulation using the quartz crystal microbalance. J. Biomech..

[18] Guhr G., Brunig R., Schmidt H., Gehrisch S., Siegert G., Weihnacht M. Monitoring changes of viscoelasticity during blood coagulation with acoustic sensors. Proceedings of the IEEE International Frequency Control Symposium, 2007 Joint with the 21st European Frequency and Time Forum.

[19] Si S.H., Xu Y.J., Nie L.H., Yao S.Z. (1996). Bulk acoustic wave sensor for investigating hemorheological characteristics of plasma and its coagulation. J. Biochem. Biophys. Methods.

[20] Cavic B.A., Freedman J., Morel Z., Mody M., Rand M.L., Stone D.C., Thompson M. (2001). Blood platelet adhesion to protein studied by on-line acoustic wave sensor. Analyst.

[21] Ergezen E., Appel M., Shah P., Kresh J.Y., Lec R.M., Wootton D.M. (2007). Real-time monitoring of adhesion and aggregation of platelets using thickness shear mode (TSM) sensor. Biosens. Bioelectron..

[22] Sinn S., Muller L., Drechsel H., Wandel M., Northoff H., Ziemer G., Wendel H.P., Gehring F.K. (2010). Platelet aggregation monitoring with a newly developed quartz crystal microbalance system as an alternative to optical platelet aggregometry. Analyst.

[23] Kawakami K., Harada Y., Sakasita M., Nagai H., Handa M., Ikeda Y. (1993). A new method for continuous measurement of platelet adhesion under flow conditions. ASAIO J..

[24] Hussain M., Sinn S., Zeilinger M., Northoff H., Lieberzeit P.A., Gehring F.K. (2013). Blood coagulation thromboplastine time measurements on a nanoparticle coated quartz crystal microbalance biosensor in excellent agreement with standard clinical methods. J. Biosens. Bioelectron..

[25] Hussain M., Northoff H., Gehring F.K. (2015). Dqcm beating the standard coagulometer in the domain of sensitivity range and information for hemostasis of human plasma. Biosens. Bioelectron..

[26] Guhr G., Kunze R., Martin G., Schmidt H., Weihnachr M., Gehrisch S., Siegert G. Monitoring Blood Coagulation with QCM and Sh-Saw Sensors. Proceedings of the 2005 IEEE Ultrasonics Symposium.

[27] Guhr G., Brunig R., Schmidt H., Weihnacht M., Gehrisch S., Siegert G. Surface acoustic wave resonators as novel tools for multiparametric blood analysis. Proceedings of the 2010 Annual International Conference of the IEEE Engineering in Medicine and Biology Society (EMBC).

[28] Andersson M., Sellborn A., Fant C., Gretzer C., Elwing H. (2002). Acoustics of blood plasma on solid surfaces. J. Biomater. Sci. Polym. Ed..

[29] Ehmann H.M., Mohan T., Koshanskaya M., Scheicher S., Breitwieser D., Ribitsch V., Stana-Kleinschek K., Spirk S. (2014). Design of anticoagulant surfaces based on cellulose nanocrystals. Chem. Commun..

[30] Jin J., Jiang W., Yin J., Ji X., Stagnaro P. (2013). Plasma proteins adsorption mechanism on polyethylene-grafted poly(ethylene glycol) surface by quartz crystal microbalance with dissipation. Langmuir.

[31] Aizawa H., Kurosawa S., Tozuka M., Park J.-W., Kobayashi K. (2004). Rapid detection of fibrinogen and fibrin degradation products using a smart qcm-sensor. Sens. Actuators B Chem..

[32] Doliska A., Ribitsch V., Stana Kleinschek K., Strnad S. (2013). Viscoelastic properties of fibrinogen adsorbed onto poly(ethylene terephthalate) surfaces by qcm-d. Carbohydr. Polym..

[33] Jung H., Tae G., Kim Y.H., Johannsmann D. (2009). Change of viscoelastic property and morphology of fibrin affected by antithrombin iii and heparin: Qcm-z and afm study. Colloids Surf. B Biointerfaces.

[34] Weber N., Pesnell A., Bolikal D., Zeltinger J., Kohn J. (2007). Viscoelastic properties of fibrinogen adsorbed to the surface of biomaterials used in blood-contacting medical devices. Langmuir.

[35] Hemmersam A.G., Foss M., Chevallier J., Besenbacher F. (2005). Adsorption of fibrinogen on tantalum oxide, titanium oxide and gold studied by the qcm-d technique. Colloids Surf. B Biointerfaces.

[36] Berglin M., Pinori E., Sellborn A., Andersson M., Hulander M., Elwing H. (2009). Fibrinogen adsorption and conformational change on model polymers: Novel aspects of mutual molecular rearrangement. Langmuir.

[37] Jung H., Kim J.Y., Kim Y., Tae G., Kim Y.H., Johannsmann D. (2009). Qcm and afm analysis of anticoagulant activities of sulfonated polymers against fibrin formation. Langmuir.

[38] Lakshmanan R.S., Efremov V., Cullen S.M., Killard A.J. (2014). Measurement of the evolution of rigid and viscoelastic mass contributions from fibrin network formation during plasma coagulation using quartz crystal microbalance. Sens. Actuators B Chem..

[39] Yao C., Qu L., Fu W. (2013). Detection of fibrinogen and coagulation factor viii in plasma by a quartz crystal microbalance biosensor. Sensors.

[40] Müller L., Sinn S., Drechsel H., Ziegler C., Wendel H.P., Northoff H., Gehring F.K. (2010). Investigation of prothrombin time in human whole-blood samples with a quartz crystal biosensor. Anal. Chem..

[41] Thurston G.B. (1972). Viscoelasticity of human blood. Biophys. J..

[42] Lakshmanan R.S., Efremov V., Cullen S., Byrne B., Killard A.J. Monitoring the effects of fibrinogen concentration on blood coagulation using quartz crystal microbalance (QCM) and its comparison with thromboelastography. Proceedings of the Bio-MEMS and Medical Microdevices.

[43] Banfi G., Del Fabbro M. (2008). Biological variation in tests of hemostasis. Semin. Thromb. Hemost..

[44] Bioanalytik R.F. Referenzinstitut für Bioanalytik. http://www.dgkl-rfb.de/4daction/g_show_plotNEW/00000000000000000000GR1121_052900.

[45] Lowe G.D., Rumley A., Mackie I.J. (2004). Plasma fibrinogen. Ann. Clin. Biochem..

[46] MDA (1999). Medical Devices Agency Evaluation Report: Fibrinogen Standards and Reference Preparations.

[47] MDA (2000). Medical Devices Agency Evaluation Report: Fibrinogen Assay Reagent and Methods.

[48] Van Den Besselaar A.M.H.P., Haas F.J.L.M., Van Der Graaf F., Kuypers A.W.H.M. (2009). Harmonization of fibrinogen assay results: Study within the framework of the dutch project “calibration 2000”. Int. J. Lab. Hematol..

[49] Marbet G.A., Duckert F., Jespersen J., Bertina R.M., Haverkate F. (1992). Fibrinogen. Ecat Assay Procedures: A Manual of Laboratory Techniques.

[50] Mackie J., Lawrie A.S., Kitchen S., Gaffney P.J., Howarth D., Lowe G.D., Martin J., Purdy G., Rigsby P., Rumley A. (2002). A performance evaluation of commercial fibrinogen reference preparations and assays for clauss and pt-derived fibrinogen. Thromb. Haemost..

